# Giant Morgagni hernia with transthoracic herniation of the left liver lobe and transverse colon: a case report

**DOI:** 10.1186/s13256-023-03914-0

**Published:** 2023-04-24

**Authors:** Osama Albasheer, Nasser Hakami, Anas A. Ahmed

**Affiliations:** 1 Family and Community Medicine Department, Faculty of Medicine, Jazan University, Jazan, Saudi Arabia; 2 Surgical Department, Faculty of Medicine, Jazan University, Jazan, Saudi Arabia

**Keywords:** Case report, Morgagni hernia, Congenital defect, Abdominal pain, Vomiting

## Abstract

**Background:**

A Morgagni hernia is a rare diaphragmatic hernia that is usually asymptomatic but can present with gastrointestinal and chest symptoms and is reported in many cases with strangulation. Here we report a rare case of a Morgagni hernia with transthoracic herniation of the left lobe of the liver and transverse colon that presented with abdominal pain.

**Case presentation:**

A 54-year-old Saudi female presented with abdominal pain, vomiting, and shortness of breath. Chest radiography revealed an air-containing viscus and a wide mediastinum. Computed tomography confirmed the presence of a right-sided Morgagni hernia. Reduction of the defect contents and repair of the hernia together with cholecystectomy were successfully performed using the laparoscopic approach. The patient recovered smoothly with complete resolution of preoperative symptoms.

**Conclusion:**

A Morgagni hernia is a rare diaphragmatic defect with an increased risk of incarceration. In addition to the omentum, transverse colon, and small bowel, the defect may involve the left lobe of the liver. Surgical repair is recommended in all cases of Morgagni hernia, to avoid the risk of incarceration.

## Introduction

Morgagni hernia is rare in adults and accounts for 3–5% of diaphragmatic hernias [1]. It is a congenital defect first described by Morgagni in 1769, with herniation of the abdominal contents into the thorax via a subcostosternal defect. Other types of diaphragmatic hernias include Bochdalek hernia and central tendon defects of the diaphragm [2]. Simultaneous Morgagni hernia and other nontraumatic diaphragmatic hernias are rare, and only a few cases have been reported in the literature [3]. The hernia defect was found in the anterior aspect of the diaphragm. Omental fat constitutes the majority of the hernia sac. However, the hernia sac may contain almost any intraabdominal organ, including the transverse colon (60%), stomach (12%), and small intestine [4, 5]. Liver is rarely included in the hernia sac [6]. Morgagni hernia is usually asymptomatic but may cause characteristic symptoms of chest pain, abdominal pain, and regurgitation [7]. Laparoscopic surgical repair is recommended, even in asymptomatic individuals, to avoid the risk of strangulation of hernia contents [7, 8]. In this case, we described the clinical presentation and surgical procedure of a patient with giant Morgagni hernia containing the left lobe of the liver and transverse colon.

## Case presentation

**Fig. 1:**
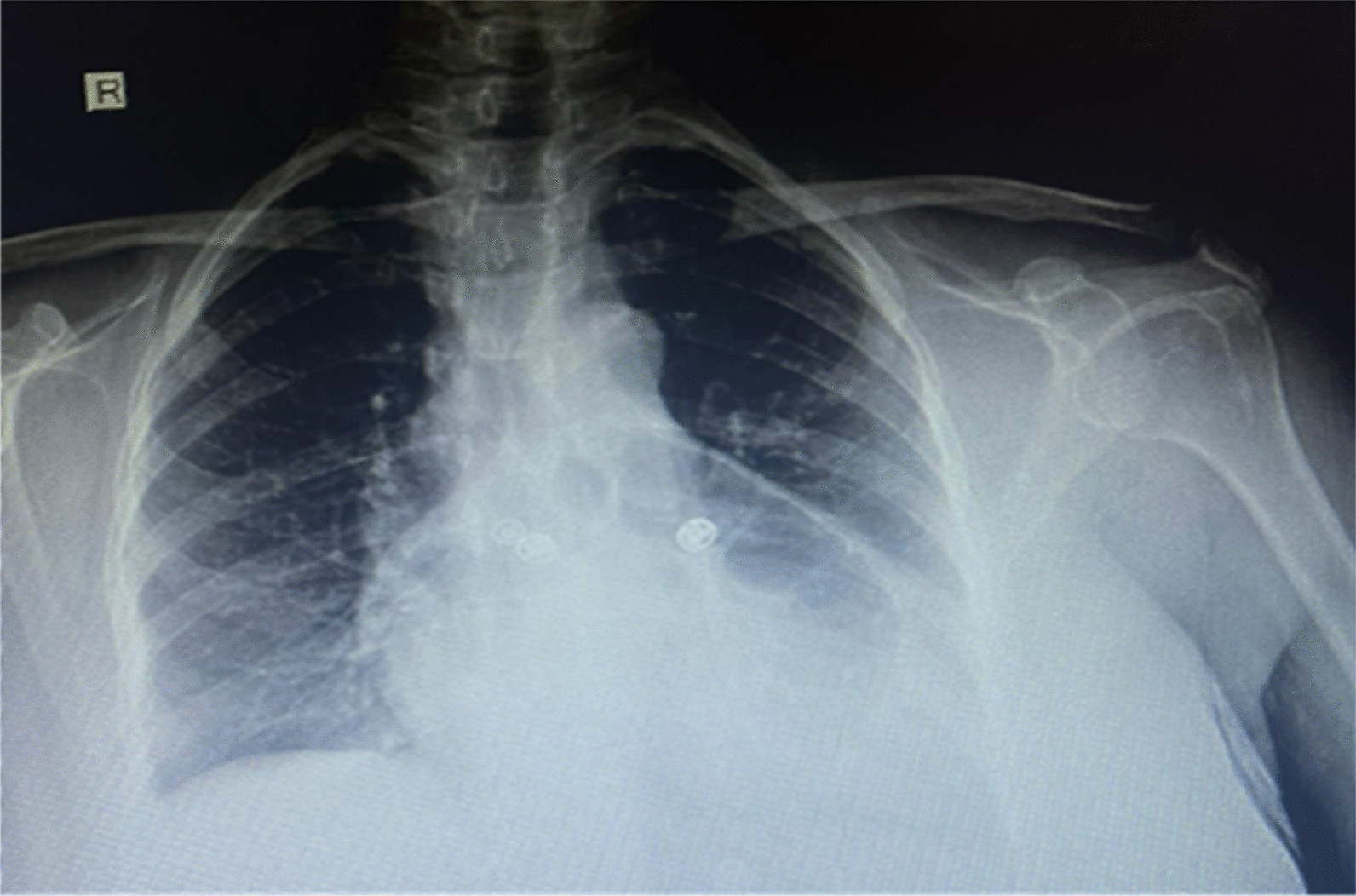
Preoperative chest radiography with a wide mediastinum and an air-containing viscus indicating intrathoracic intestine loops

**Fig. 2:**
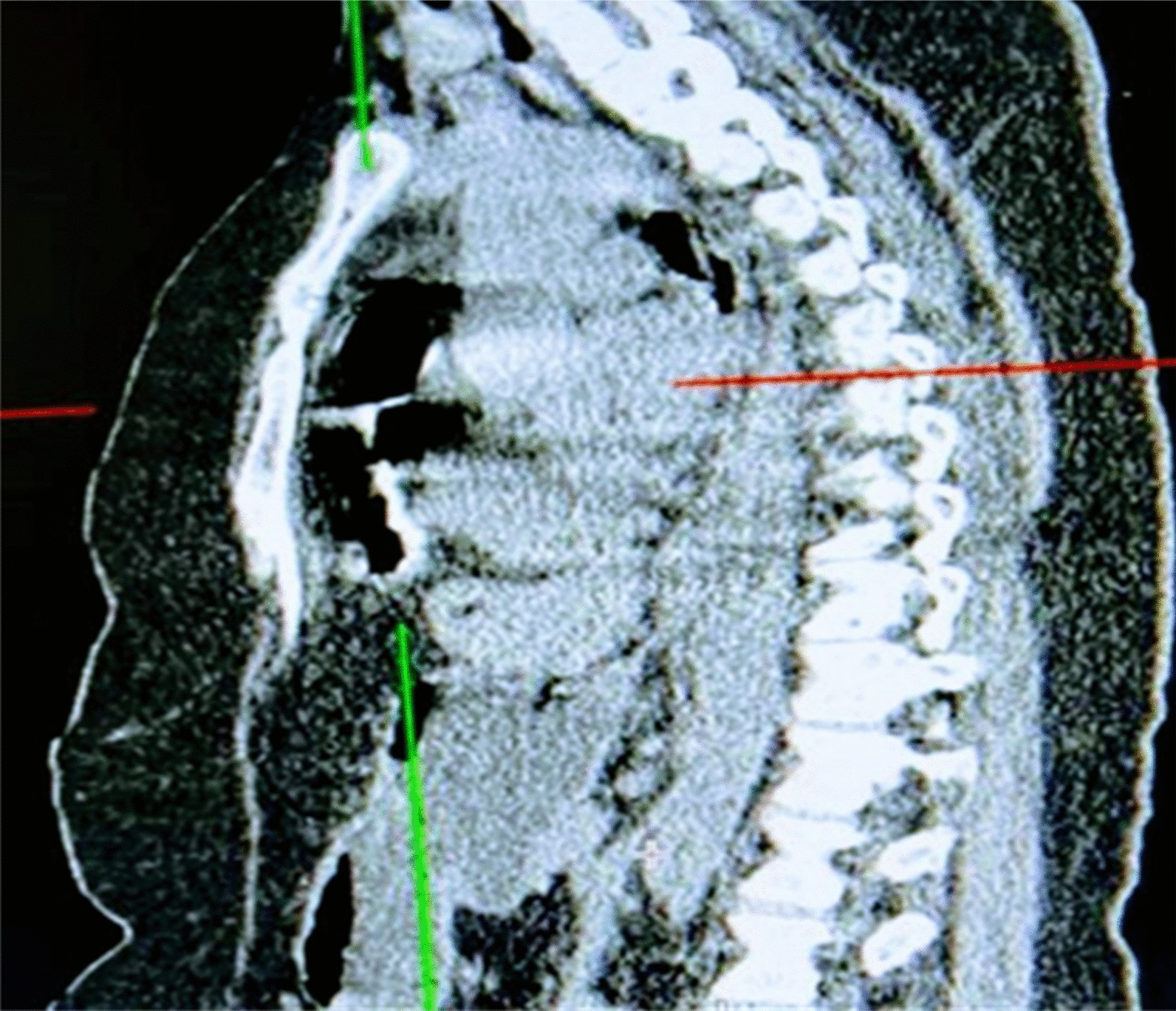
Computed tomography (CT) sagittal view showing a retrosternal mass of fat density and an air-containing viscus consistent with the presence of omentum and transverse colon, respectively

A 54-year-old Saudi female with hypertension presented with shortness of breath for the past 1 month and abdominal pain, nausea, and vomiting for 3 days. The patient is taking oral amlodipine 5 mg daily for her hypertension, which is well controlled. The abdominal pain was mild and associated with abdominal distension, particularly in the upper right quadrant. There was no constipation. The patient had a history of gallstones, treated conservatively. Apart from history of hypertension and a symptomatic gallstones, other past medical, social, environmental, and family histories were unremarkable. The patient is a housewife, living with her family, and does not smoke or consume alcohol. Physical examination revealed a moderately distressed patient with a temperature of 36.3 °C, pulse of 94 beats per minute, respiratory rate of 22 breaths per minute, blood pressure of 158/98 mmHg, body mass index of 28 kg/m^2^, and oxygen saturation of 95%. Chest auscultation revealed reduced air entry over the anterior lung field toward the bottom and laterally to the right side. The abdomen was distended and nontender with no guarding. Bowel sounds were audible. There were no other findings on physical and neurological examinations. Chest radiography revealed an air-containing viscus with a wide mediastinum (Fig. 1). Computed tomography (CT) without contrast showed an anterior midline diaphragmatic defect measuring approximately 6.5 cm with herniation of the omentum, middle part of the transverse colon, and upper part of the left lobe of the liver into the thoracic cavity, anterior to the heart, and great vessels (Fig. 2). On the basis of the clinical and radiological findings, the patient was planned for surgery. Prior to surgery, hemoglobin was 13% with a hematocrit of 42%. Liver enzymes and renal function tests were within normal limits. The patient used the usual dose of amlodipine 8 hours before the surgery and then continued on intravenous amlodipine titrated according to her weight during and after the surgery, then resumed the usual oral dose. The operating room staff monitored the patient, and the laparoscopic procedures have been performed under general anesthesia (GA).

**Fig. 3:**
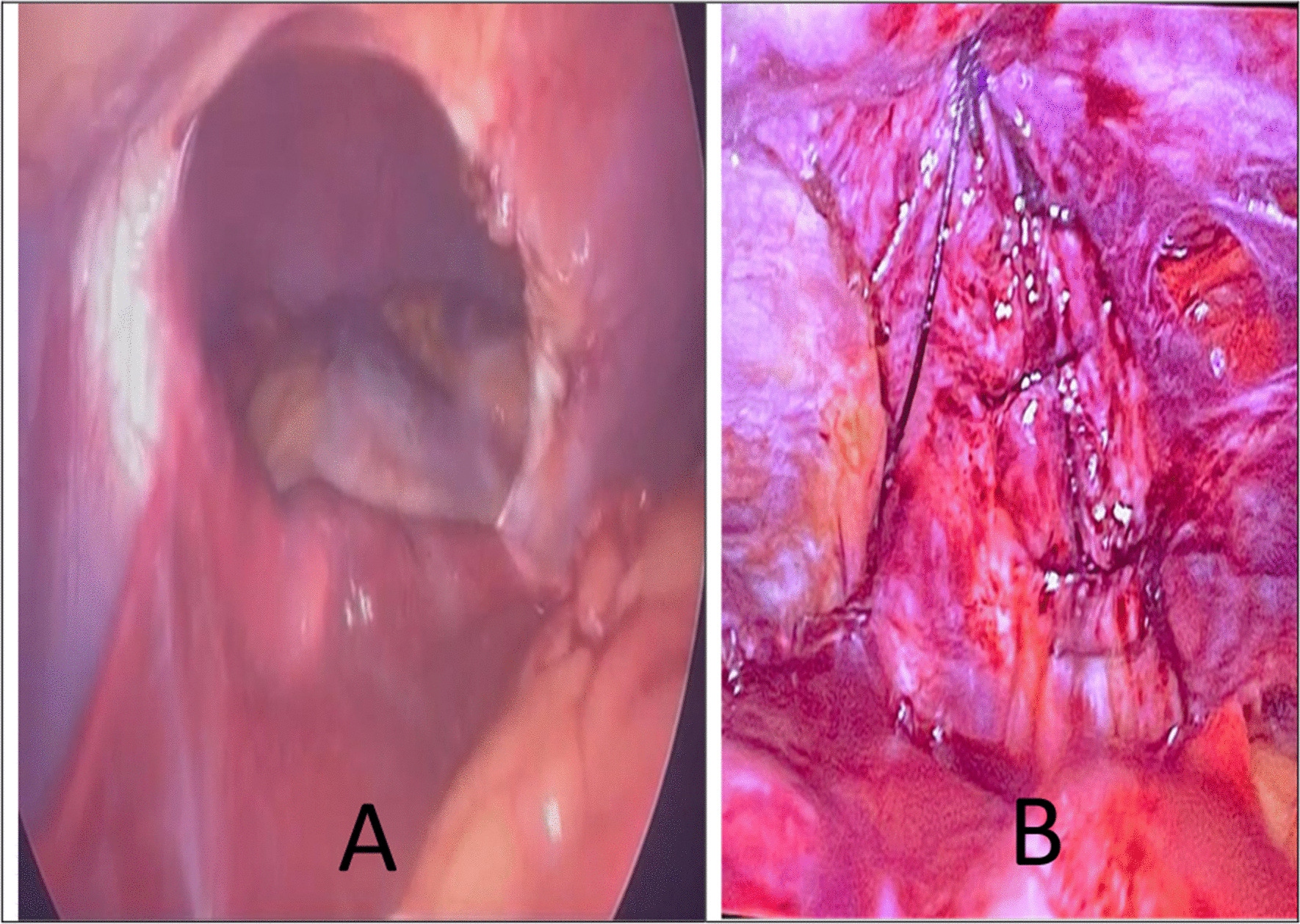
Intraoperative laparoscopic view of the Morgagni hernia (**A**) showing the omentum, transverse colon, and left lobe of the liver inside the defect. Reduction of the contents and repair of the defect by primary stitches (**B**)

The hernial defect was treated using an abdominal laparoscopic surgical approach. The adhesions were released, and a defect in the diaphragm was identified. The contents of the hernia were successfully reduced, including the omentum, middle part of the transverse colon, and upper part of the left lobe of the liver (Fig. 3A). Cholecystectomy was performed, and the defect was closed successfully by primary repair with stitches using Silk 2.0, and strengthened with propylene (using an Endo Close suture device) and an 11 × 11 cm mesh (Fig. 3B).

**Fig. 4:**
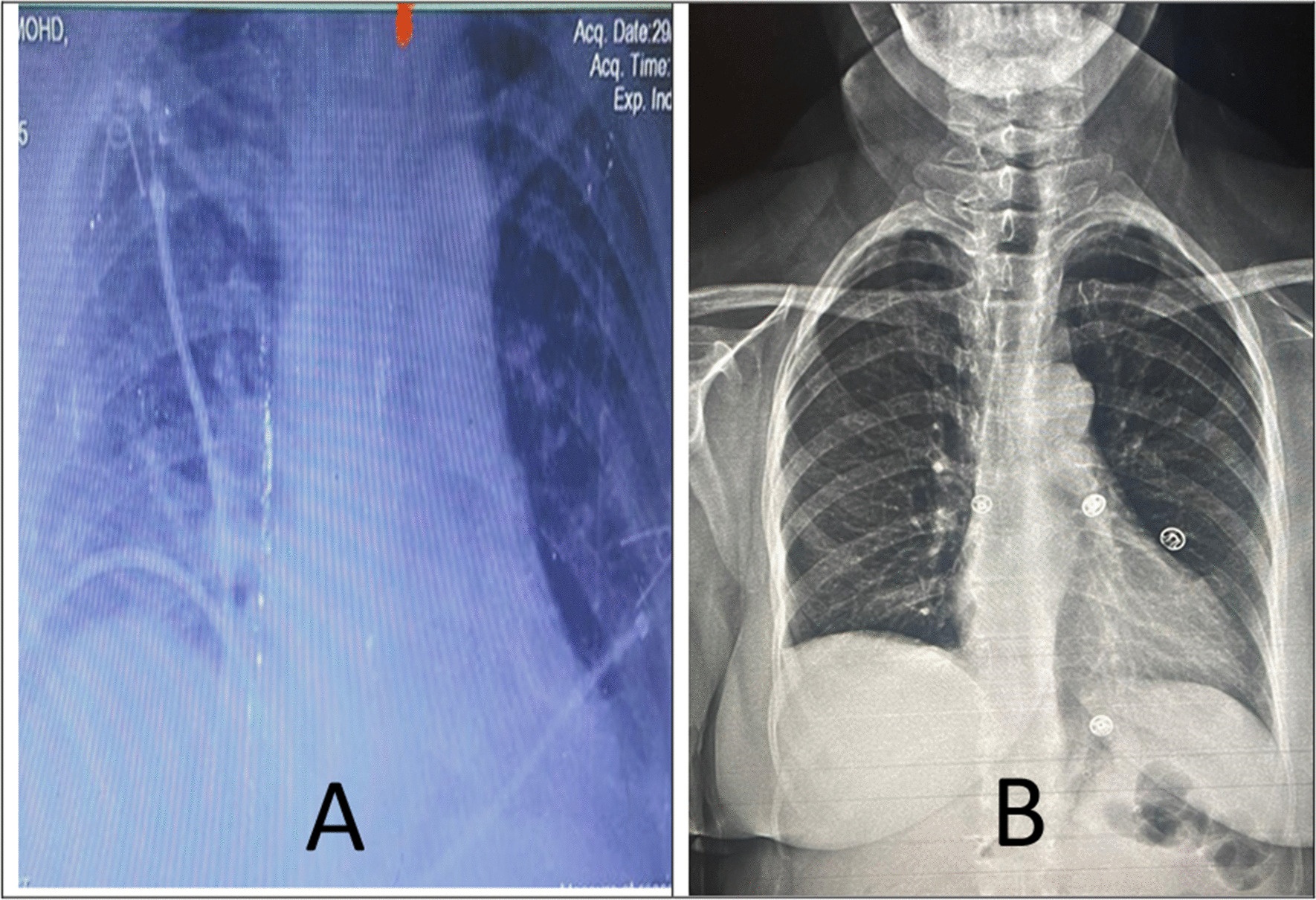
Chest radiography done on the second day postoperation showing white spot infiltration and air under the right diaphragm (**A**). Chest radiography done after 2 months showed no abnormalities (**B**)

The oxygen saturation dropped to 88% in the first 2 hours after the operation. The patient was admitted to the intensive care unit (ICU). Chest radiography revealed white spot infiltration (pneumonia) and atelectasis (Fig. 4A). Infusion of cefuroxime 750 mg was commenced and continued every 8 hours for 3 days. The patient’s oxygen saturation significantly improved (96% on 3 L of oxygen), and the lungs reflated adequately. The patient was transferred to the general ward on oral cefuroxime 500 mg two times per day and physiotherapy. The patient was discharged on the seventh postoperative day in a good condition. On first follow-up after 2 months, the patient had no complaints and was satisfied and pleased. Chest radiography revealed normal findings (Fig. 4B).

## Discussion

The main findings in this case report are the presence of huge Morgagni hernia, in an elderly female, with transthoracic herniation of the left lobe of the liver and transverse colon, presented with a combination of gastrointestinal and respiratory symptoms. The uniqueness in this patient is the subacute presentation of abdominal pain and synchronous prolapse of large part of the liver with the transverse colon, which is rarely reported in the literature [9].

Morgagni hernia is one of the three types of diaphragmatic hernias in which the defect is located posterolaterally to the sternum in the anterior and retrosternal locations [4, 5]. It is less common than other types of congenital diaphragmatic hernias (accounting for only 2–5%) [1, 3]. Bochdalek hernia is the commonest type of congenital diaphragmatic hernias [2]. Morgagni hernia in this case was on the right side. Although bilateral and left-sided hernias can develop, 90% of Morgagni hernias occur on the right side because of pericardial attachments to the diaphragm, which support and shield the left side [10].

The patient presented with shortness of breath, abdominal pain, nausea, and vomiting. Similar presentations of combined gastrointestinal and respiratory symptoms have been reported in adults, including retrosternal or chest pain, which is often relieved by standing, shortness of breath, flatulence, indigestion, and cramping [5, 7, 10–13]. Nevertheless, up to 50% of patients may not have any symptoms upon presentation, with the diagnosis occurring during the course of treatment for unrelated problems [6, 14]. Usually the symptoms of Morgagni hernia are mild to moderate in severity and could be related to the size of the defect, content of the hernia, or pressure exerted on the thoracic structures with common symptoms, including pulmonary and gastrointestinal symptoms. However, few cases of strangulated Morgagni hernia have been reported [14].

The main contents of the hernia sac in our patient were the omentum, the middle part of the transverse colon, and the upper part of the left lobe of the liver. Various abdominal organs, including the transverse colon, stomach, omentum, and small intestine, have been reported in the hernial sac; however, few cases have reported a part of the liver in the hernial sac [4, 7, 15].

The diagnosis of Morgagni hernia was suggested from the presentation, physical examination, and chest radiography, and confirmed by the computed tomography scan without contrast. Conventional radiography is usually helpful in the diagnosis of Morgagni hernia, with occasional missed diagnosis [5, 16]. Computed tomography (CT) and magnetic resonance imaging (MRI) are commonly used to confirm diagnosis and differentiate Morgagni hernias from other intrathoracic and diaphragmatic defects [14, 15].

In this case, a laparoscopic approach was used, with reduction of the hernia contents and repair of the defect using mesh and polypropylene sutures. Surgery was preferred because the patient was symptomatic with a subacute presentation of abdominal pain and vomiting. Repair is recommended, even in asymptomatic patients, to avoid incarceration [7, 8]. Some advocate conservative approaches for asymptomatic defects to avoid the risk of adhesion [14].

Most patients are usually discharged 3 days after surgery after an uneventful recovery [11, 14]. In this case, the patient’s condition was complicated by pneumonia, and she was treated properly, recovered, and discharged after 7 days without the need for postoperative ventilation.

## Conclusion

A Morgagni hernia is a rare congenital diaphragmatic defect. It is usually asymptomatic but may present in late adulthood with characteristic symptoms of chest pain, abdominal pain, and vomiting. The defect is most commonly composed of omental fat and the small and large intestines; however, it may also include the liver and other organs. Owing to the increased risk of incarceration, surgical repair is recommended in all cases of Morgagni hernia.

## Data Availability

Datasets related to this case report are available from the corresponding author upon request.

## Abbreviations

CT: Computed tomography
ICU: Intensive care unit
MRI: Magnetic resonance imaging
